# Prevalence of Chlamydia trachomatis, Neisseria gonorrhoeae, and Ureaplasma urealyticum infections among reproductive-age adults in Shantou, China: a retrospective study

**DOI:** 10.1186/s12879-026-13701-z

**Published:** 2026-05-30

**Authors:** Chusheng Huang, Lixin Chen, Fan Yang, Tongtong Xiao

**Affiliations:** 1 Department of General Surgery, Shantou Central Hospital, Guangdong, China; 2 Department of Clinical Laboratory, Shantou Central Hospital, Guangdong, China

**Keywords:** Sexually transmitted infections, Chlamydia trachomatis, Neisseria gonorrhoeae, Ureaplasma urealyticum, Childbearing age

## Abstract

Sexually transmitted infections (STIs) remain a major global public health concern. Chlamydia trachomatis (CT), Neisseria gonorrhoeae (NG), and Ureaplasma urealyticum (UU) are among the most common bacterial STI pathogens. Data on the test positivity of these pathogens in reproductive-age populations are essential for informing targeted screening and prevention strategies. This retrospective study assessed the test positivity and co-infection patterns of CT, NG, and UU among 13,622 reproductive-age outpatients in Shantou, China, between January 2023 and December 2024. Urogenital specimens were tested using nucleic acid amplification assays, and associations with clinical diagnoses were examined. Overall, 11.14% of participants tested positive for at least one pathogen; however, as not all participants were tested for all three pathogens (CT: 97.2%; NG: 53.4%; UU: 16.4%), this figure should be interpreted as a likely underestimate of the true positivity in this population. Marked sex differences were observed: CT and UU test positivity was higher in females (3.68% and 48.97%, respectively), whereas NG test positivity was higher in males (15.01% vs. 0.58%). By age, CT and NG test positivity was higher among younger participants (18–24 and 25–30 years) in both sexes, whereas UU test positivity remained consistently high across age groups. Across clinical diagnoses, urogenital inflammation had the highest test positivity for all three pathogens, while infertility-related diagnoses had comparatively lower test positivity. These findings provide evidence to inform targeted STI screening and prevention strategies to protect reproductive health in coastal China.

## Introduction

Sexually transmitted infections (STIs) continue to be a significant global public health issue, severely affecting reproductive health and fertility worldwide [1–3]. According to the World Health Organization (2024), more than one million curable STIs are acquired daily by individuals aged 15–49 years, with Chlamydia trachomatis (CT), Neisseria gonorrhoeae (NG), and Ureaplasma urealyticum (UU) being the most prevalent bacterial pathogens. In 2020, it was estimated that 129 million new CT infections and 82 million NG infections occurred globally, alongside substantial rates of UU infections, the majority of which remain asymptomatic and undiagnosed [4].

CT, NG, and UU infections significantly contribute to reproductive morbidity through distinct pathogenic mechanisms. CT, an obligate intracellular pathogen, causes urethritis, cervicitis, and pelvic inflammatory disease (PID), leading to complications such as tubal infertility, ectopic pregnancy, and chronic pelvic pain in 10–15% of untreated women [5]. NG typically presents as urethritis in men and cervicitis or urethritis in women. It can also affect extragenital sites, such as the pharynx, rectum, and conjunctiva, occasionally leading to systemic dissemination [6]. In 2002, Ureaplasma was officially separated into two species, U. urealyticum and U. parvum, which differ phenotypically and genotypically, with potential pathogenicity attributed more to U. urealyticum, whereas U. parvum is considered a commensal organism [7, 8]. UU infection can affect the morphology and quality of male sperm and is therefore an important cause of male infertility [9]. UU and CT are important pathogens in nongonococcal urethritis. Furthermore, STIs increase susceptibility to HIV infection by two- to fivefold through mucosal inflammation [10]. Co-infections involving CT, NG, and UU are common and may synergistically exacerbate inflammation, accelerate disease progression, and complicate clinical management [11, 12]. The cumulative effects on fertility, pregnancy outcomes, and HIV transmission highlight the urgent need for comprehensive epidemiological studies.

Individuals of reproductive age are particularly vulnerable to STI acquisition and its associated complications, exhibiting distinct epidemiological characteristics that warrant focused investigation [13]. In China, demographic shifts—including relaxed family planning policies and delayed childbearing—have made understanding STI epidemiology in this population increasingly critical for protecting reproductive potential and preventing vertical transmission. Current epidemiological data on STIs among individuals of reproductive age in China remain limited. However, surveillance data from Chengdu, China, indicate that the overall prevalence of CT, UU, and NG is 5.06%, 41.00%, and 0.52% among women, and 1.47%, 7.41%, and 0.11% among men, respectively [14]. Shantou, a coastal city with over five million residents, lacks comprehensive STI surveillance data despite its socioeconomic significance and demographic diversity.

Therefore, this retrospective study of 13,622 outpatients in Shantou aims to investigate the prevalence and co-infection patterns of CT, NG, and UU, as well as their associations with clinical diagnoses. The findings provide valuable evidence to inform targeted STI screening and prevention strategies, ultimately supporting improvements in reproductive health in China’s coastal regions.

## Materials and methods

### Study population

This retrospective cross-sectional study was conducted from January 1, 2023, from December 31, 2024, at Shantou Central Hospital. A total of 13,622 participants of childbearing age were enrolled, comprising 3,618 males (mean age, 32.75 years) and 10,004 females (mean age, 32.48 years). The inclusion criteria were as follows: (a) residents of Shantou aged 18–55 years; (b) abstinence from sexual activity for at least 24 h before specimen collection; (c) no previous testing for CT, UU, or NG within the preceding 12 months(to avoid including patients undergoing repeat or follow-up testing); and (d) inclusion of pregnant and lactating women. The exclusion criteria were: (a) incomplete demographic or clinical data, such as missing or erroneous identification number, absence of clinical diagnosis, missing age or sex information, or unspecified specimen type; (b) repeated testing for CT, NG, or UU after the initial screening; (c) use of vaginal medications or systemic antibiotics within the preceding two weeks; (d) non-genitourinary specimens; and (e) age outside the range of 18–55 years.

Among the 3,618 males screened, 3,516 specimens were obtained for CT testing, 676 for UU testing, and 573 for NG testing. Among the 10,004 females screened, 9,725 specimens were obtained for CT testing, 1,554 for UU testing, and 6,705 for NG testing. Testing strategy was clinician-directed based on clinical presentation, patient concerns, and risk assessment. All participants underwent at least one pathogen test, but not all were tested for all three pathogens. The total number of individuals tested, and the number of CT, UU, and NG tests performed among males and females of childbearing age in different age groups are presented in Table 1.

**Table 1:** Total number of individuals tested and the number of CT, UU and NG tests performed among males and females of childbearing age across different age groups in Shantou, 2023–2024

Age groups (years)	Males				Females			
	Total test(*n*)	CT test(*n*)	UU test(*n*)	NG test(*n*)	Total test(*n*)	CT test(*n*)	UU test(*n*)	NG test(*n*)
18–24	272	169	43	60	1128	607	83	438
25–30	1346	1001	195	150	7247	3888	355	3004
31–35	1682	1316	199	167	5211	2817	337	2057
36–45	1092	808	158	126	3303	1801	491	1011
46–55	373	222	81	70	1095	612	288	195
Total	4765	3516	676	573	17,984	9725	1554	6705

### Specimen and clinical data collection

Genital swabs (Medicon Technology Co., Ltd., Shenzhen, China) were used to collect secretions from male genital sites (glans, prepuce, and urethral meatus) and from the female vaginal introitus. All specimens were collected by experienced physicians. After collection, each swab was placed into a sterile tube and transported to the laboratory for testing. The specimens were transported to the molecular biology laboratory within 2 h of collection and stored in a dedicated refrigerator at 2–8 °C until further processing. All samples were tested within 24 h of collection. Demographic and clinical data were obtained from the patients’ electronic medical records in the hospital information system.

### CT, UU and NG pathogens detection

CT, UU, and NG qPCR kits(Sansure Biotech Inc., China) were used to detect the presence of pathogens. All operations were strictly performed in accordance with the manufacturer’s instructions. All tests were completed on the LightCycler 480 II Real-Time PCR instrument (Roche Diagnostic, Shanghai, China). Negative control and positive control were performed in each assay (Sansure Biotech Inc., China). Positive UU (CT or NG) diagnostic criteria were as follows: Cycle threshold value ≤ 38 was diagnosed as positive, and cycle threshold value > 38 was diagnosed as negative. Clinical and Laboratory Standards Institute guidelines were used to ensure the accuracy of the testing results. At the same time, the testing is included in the external quality assessment (EQA), which is usually organized by National Center for Clinical Laboratory (NCCL) of China (twice every year). The results of EQA indicate that the quality of products from this company should be reliable. The limit of detection of this assay is 400 copies/mL.

### Statistical analysis

Data were cleaned and managed in Microsoft Excel 2021. The χ² test was applied to compare positivity rates between groups. Univariate logistic regression analyses were performed to evaluate the associations between pathogen positivity (CT, NG, and UU) and variables including age group and clinical diagnosis; odds ratios (ORs) with 95% confidence intervals (95% CIs) were calculated, and statistical significance was assessed using the Wald χ² test with corresponding p-values. SPSS Statistics 20.0 (SPSS Inc., Chicago, IL, USA) was used for all statistical analyses. A two-tailed p-value of < 0.05 was considered statistically significant. Graphical presentations were generated using Origin 2021 (OriginLab Corporation, Northampton, MA, USA) and R version 4.2.3.

## Results

### Positivity rates of CT, NG, and UU infections in males and females of childbearing age

From January 2023 to December 2024, a total of 13,622 patients were included in this study, comprising 3,618 males (26.56%) and 10,004 females (73.44%). The mean age of participants was 32.75 ± 6.77 years. Not all participants underwent testing for all three pathogens due to selective clinical ordering: 13,241 (97.2%) were tested for CT, 7,278 (53.4%) for NG, and 2,230 (16.4%) for UU. Overall, 1,517 participants (11.14% of the total cohort) tested positive for at least one pathogen. Pathogen-specific positivity rates among tested individuals were: UU 42.42% (946/2,230), CT 4.16% (551/13,241), and NG 1.72% (125/7,278). The overall positivity rate did not differ significantly between sexes: 1,103 of 10,004 females (11.03%) and 414 of 3,618 males (11.44%) tested positive for at least one pathogen (χ² = 0.33, *p* = 0.56).

**Fig. 1:**
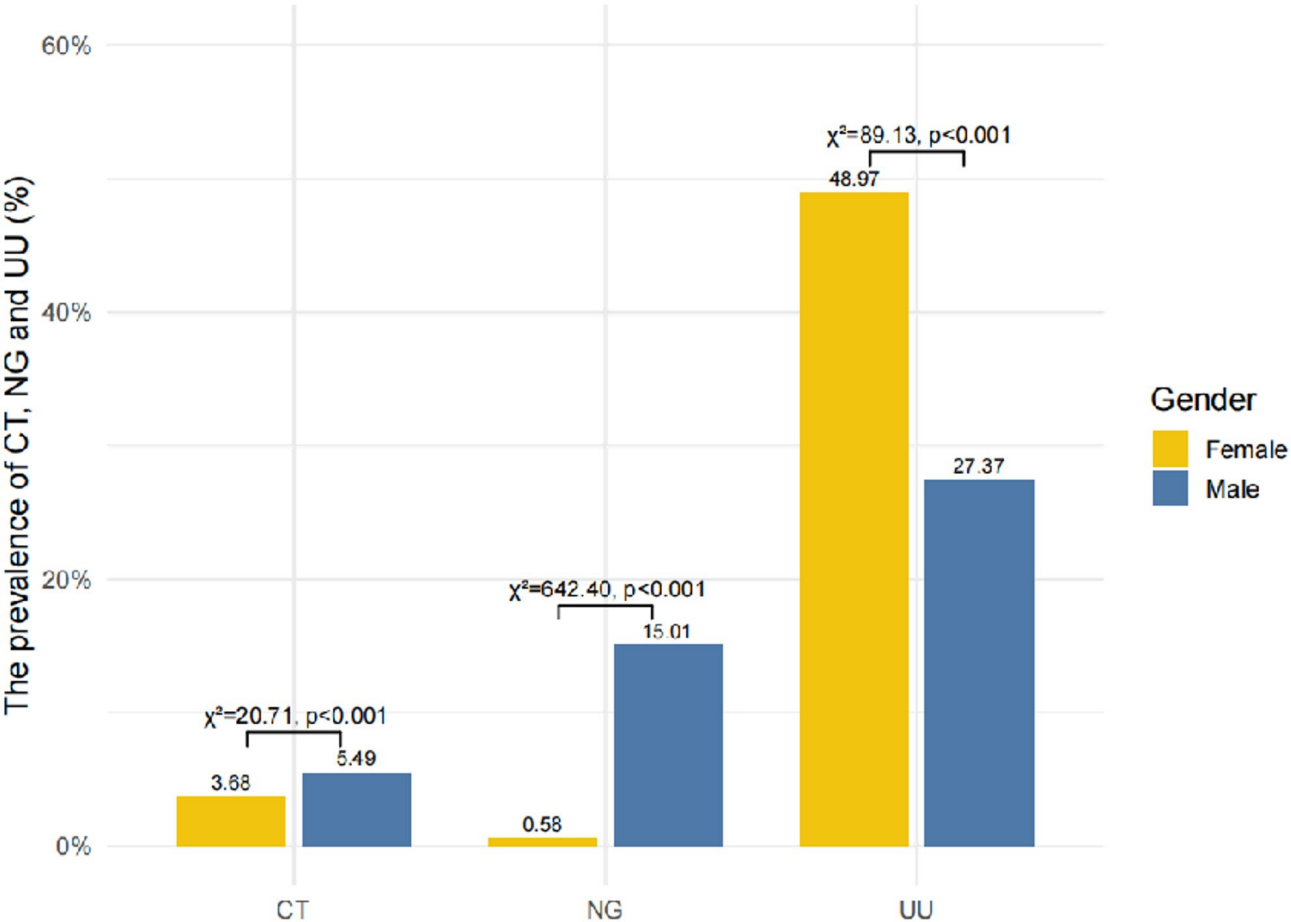
Positivity rates of CT, NG and UU infections in both males and females of childbearing age (%)

However, pathogen-specific prevalence showed significant sex-based differences (Fig. 1). Among those tested, CT and UU prevalence were significantly higher in females than in males (both *p* < 0.05), whereas NG prevalence was significantly higher in males (*p* < 0.05).

### Co-infection analysis among the three pathogens

**Fig. 2:**
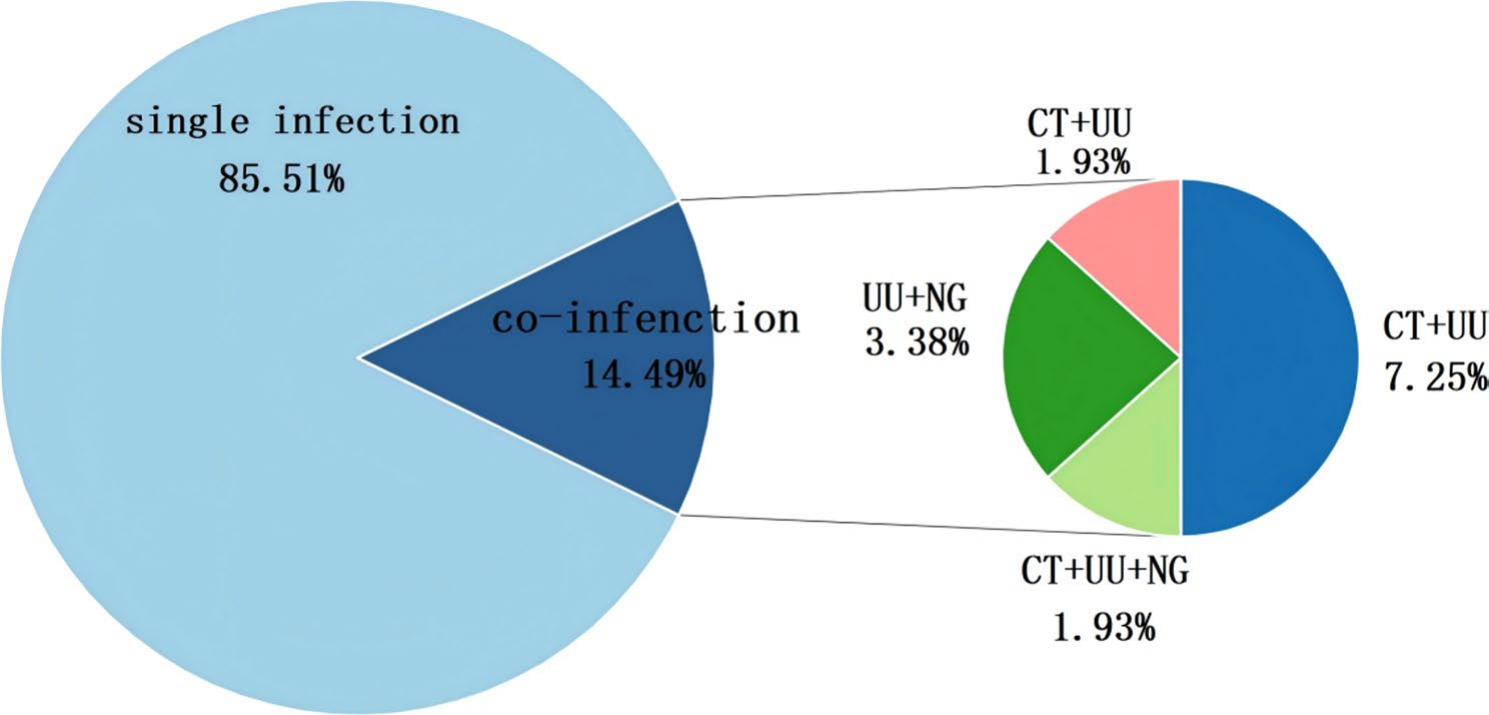
Distribution of infection patterns (single infection vs. co-infection) and co-infection combinations among 3,657 patients tested for CT, UU, and NG

To characterize co-infection patterns, we limited the analysis to patients tested for all three pathogens (*n* = 3,657). As shown in Fig. 2, co-infections were detected in 0.82% of patients (30/3,657; 95% CI: 0.58%–1.17%). Among pathogen-positive patients (*n* = 207; 5.66%, 95% CI: 4.96%–6.46%), co-infections accounted for 14.49% (30/207). The most common co-infection pattern was CT + UU (50.00%), followed by UU + NG (23.33%); triple infection (CT + UU+NG) accounted for 13.33% of co-infections. UU was the pathogen most frequently involved in co-infections, with UU-containing combinations comprising 86.67% of co-infected cases.

### Comparison of positive rates of CT, NG, and UU in different age groups

**Fig. 3:**
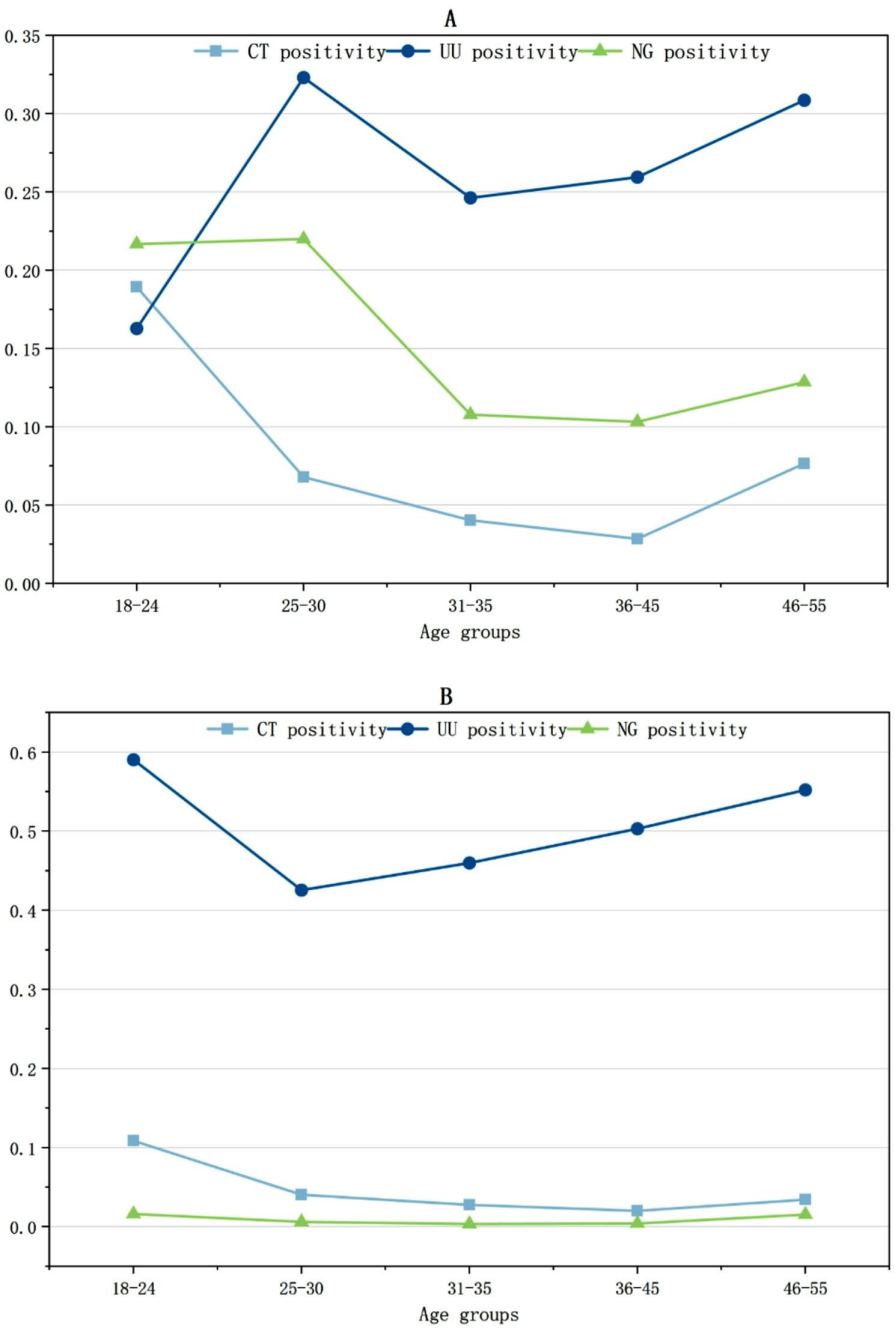
The positive rate of various pathogens in different age groups. (**A**) Male participants. (**B**) Female participants

The positive rates of CT, NG, and UU across age groups for both sexes is summarized in Table 2; Fig. 3. In males, CT and NG positive rates varied significantly among age groups (*p* < 0.05), with the highest rates in younger age groups (18–24 and 25–30 years). UU positive rate peaked in the 25–30 year group but did not differ significantly across age groups (*p* > 0.05).

In females, significant age-related differences were observed for all three pathogens (*p* < 0.05). CT positive rate was highest among those aged 18–24 years and declined with age. UU positive rate remained consistently high, peaking at 31–35 years, while NG positive rate was relatively low overall but showed slight increases in the 18–24 and 46–55 year groups.

**Table 2:** Comparison of positive rates for various pathogens across age groups in males and females

Age groups (years)	Males			Females		
	CT positivity, *n*/*N* (%)	UU positivity, *n*/*N* (%)	NG positivity, *n*/*N* (%)	CT positivity, *n*/*N* (%)	UU positivity, *n*/*N* (%)	NG positivity, *n*/*N* (%)
18–24	32/169 (18.93%)	7/43 (16.28%)	13/60 (21.67%)	66/607 (10.87%)	49/83 (59.04%)	7/438 (1.60%)
25–30	68/1001 (6.79%)	63/195 (32.31%)	33/150 (22.00%)	157/3888 (4.04%)	151/355 (42.54%)	18/3004 (0.60%)
31–35	53/1316 (4.03%)	49/199 (24.62%)	18/167 (10.78%)	78/2817 (2.77%)	155/337 (45.99%)	7/2057 (0.34%)
36–45	23/808 (2.85%)	41/158 (25.95%)	13/126 (10.32%)	36/1801 (2.00%)	247/491 (50.31%)	4/1011 (0.40%)
46–55	17/222 (7.66%)	25/81 (30.86%)	9/70 (12.86%)	21/612 (3.43%)	159/288 (55.21%)	3/195 (1.54%)
χ²	81.10	6.47	12.60	111.04	15.28	13.61
P	< 0.05	> 0.05	< 0.05	< 0.05	< 0.05	< 0.05

In univariable logistic regression analyses using the 18–24-year age group as the reference, CT positivity among reproductive-aged males was significantly lower in all older age groups (OR range 0.13–0.36; all *p* ≤ 0.001). For UU, only men aged 25–30 years had higher odds than the reference group (OR = 2.46, 95% CI 1.04–5.82; *p* = 0.042), whereas no significant differences were observed for other age groups (all *p* ≥ 0.082). For NG, odds were significantly lower in men aged 31–35 years (OR = 0.44, 95% CI 0.20–0.96; *p* = 0.039) and 36–45 years (OR = 0.42, 95% CI 0.18–0.96; *p* = 0.041), with no significant differences observed in the 25–30 or 46–55-year groups (Table 3). Among females, CT positivity was highest in those aged 18–24 years (10.87%) and decreased significantly with increasing age. Compared with the reference group, women aged 25–30 (OR = 0.34), 31–35 (OR = 0.23), 36–45 (OR = 0.17), and 46–55 years (OR = 0.29) had significantly lower odds of CT positivity (all *p* < 0.001). UU positivity was also highest among women aged 18–24 years (59.04%); relative to this group, odds were significantly lower in women aged 25–30 (OR = 0.51, *p* = 0.007) and 31–35 years (OR = 0.59, *p* = 0.034), while differences were not statistically significant for women aged 36–45 or 46–55 years. Overall, NG positivity was low among females (0.34%–1.60%). Women aged 25–45 years had significantly lower odds of NG positivity than those aged 18–24 years, whereas no significant difference was observed for women aged 46–55 years, with wide confidence intervals indicating imprecision (Table 4).

**Table 3:** Univariable logistic regression analysis of the associations between age and CT, NG, and UU positivity among males of reproductive age

Age group (years)	CT				UU				NG			
	Odds ratio	95% CI	Wald	*p*-value	Odds ratio	95% CI	Wald	*p*-value	Odds ratio	95% CI	Wald	*p*-value
18–24	1	—	—	—	1	—	—	—	1	—	—	—
25–30	0.31	0.20–0.49	24.97	< 0.001	2.46	1.04–5.82	4.15	0.042	1.02	0.49–2.11	0	0.958
31–35	0.18	0.11–0.29	50.63	< 0.001	1.68	0.70–4.02	1.36	0.243	0.44	0.20–0.96	4.28	0.039
36–45	0.13	0.07–0.22	51.73	< 0.001	1.8	0.74–4.36	1.7	0.192	0.42	0.18–0.96	4.18	0.041
46–55	0.36	0.19–0.66	10.49	0.001	2.3	0.90–5.86	3.02	0.082	0.53	0.21–1.35	1.75	0.186

**Table 4:** Univariable logistic regression analysis of the associations between age and CT, NG, and UU positivity among females of reproductive age

Age group (years)	CT				UU				NG			
	Odds ratio	95% CI	Wald	*p*-value	Odds ratio	95% CI	Wald	*p*-value	Odds ratio	95% CI	Wald	*p*-value
18–24	1	—	—	—	1	—	—	—	1	—	—	—
25–30	0.34	0.26–0.47	47.93	< 0.001	0.51	0.32–0.83	7.24	0.007	0.37	0.15–0.89	4.89	0.027
31–35	0.23	0.17–0.33	70.12	< 0.001	0.59	0.36–0.96	4.48	0.034	0.21	0.07–0.60	8.43	0.004
36–45	0.17	0.11–0.25	70.55	< 0.001	0.7	0.44–1.13	2.15	0.142	0.24	0.07–0.84	5.01	0.025
46–55	0.29	0.18–0.48	22.95	< 0.001	0.86	0.52–1.40	0.38	0.536	0.96	0.25–3.76	0	0.956

### Analysis of CT, NG and UU Infections according to clinical diagnoses

**Fig. 4:**
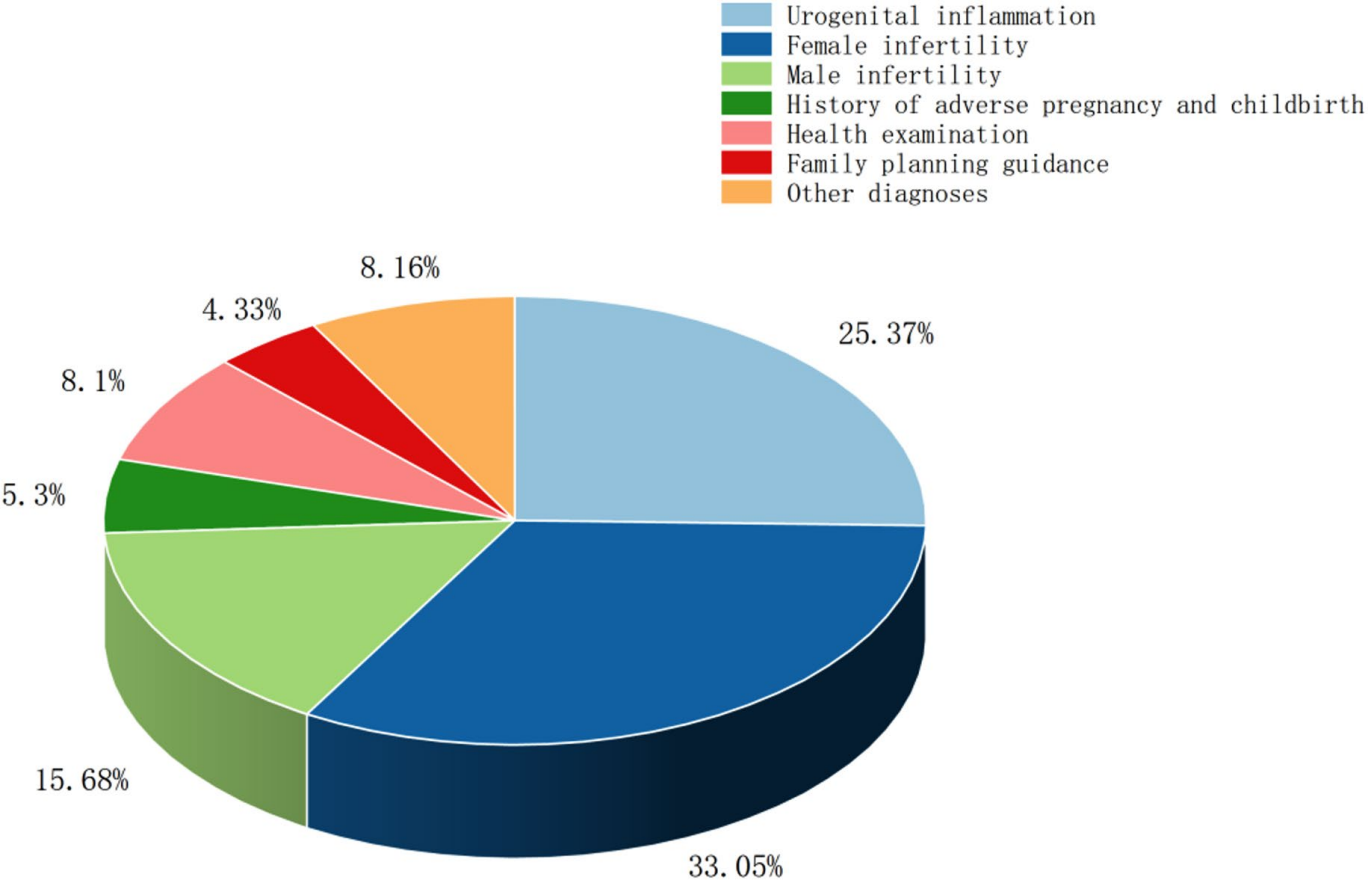
Proportion of participants across different diagnostic categories

As shown in Fig. 4, infertility represented the predominant diagnostic category among all participants, with female infertility accounting for the largest proportion, followed by urogenital inflammation and male infertility. Health examination, history of adverse pregnancy and childbirth, family planning guidance, and other diagnoses collectively comprised smaller proportions.

As summarized in Table 5, infection rates of CT, NG, and UU (calculated based on the total number of patients within each diagnostic category) differed significantly across clinical groups (all *p* < 0.05). UU infection was highest among patients with urogenital inflammation (16.75%), followed by those undergoing health examinations (11.50%). CT infection peaked among patients receiving family planning guidance (6.07%), while NG showed the highest rate in urogenital inflammation (2.63%). Notably, patients with infertility—both male and female—and those with adverse pregnancy history demonstrated consistently lower infection rates across all three pathogens.

Univariable logistic regression analysis, using routine health examination as the reference category, revealed distinct patterns of pathogen association (Table 6). CT positivity was significantly lower among patients with female infertility, male infertility, and adverse pregnancy history (all *p* ≤ 0.002). Conversely, urogenital inflammation was associated with elevated risk of both UU (OR = 1.55, 95% CI 1.26–1.90; *p* < 0.001) and NG (OR = 2.27, 95% CI 1.26–4.07; *p* = 0.006) infection.

**Table 5:** Infection rates of CT, UU, and NG infections across different clinical diagnoses

Clinical diagnoses	Number	CT(%)	UU(%)	NG(%)
Urogenital inflammation	3456	211(6.11)	579(16.75)	91(2.63)
Female infertility	4502	133(2.95)	53(1.18)	10(0.22)
Male infertility	2136	23(1.08)	22(1.03)	1(0.05)
History of adverse pregnancy and childbirth	722	20(2.77)	10(1.39)	0(0.00)
Health examination	1104	67(6.07)	127(11.50)	13(1.18)
Family planning guidance	590	42(7.12)	65(11.02)	4(0.68)
Other diagnoses	1112	49(4.41)	76(6.83)	6(0.54)
χ²		3985.4	4171.5	4328.7
P		< 0.05	< 0.05	< 0.05

**Table 6:** Univariable logistic regression analysis of the association between diagnostic categories and CT, NG, and UU positivity in a reproductive-age population

Clinical diagnoses	CT				UU				NG			
	Odds ratio	95% CI	Wald	*p*-value	Odds ratio	95% CI	Wald	*p*-value	Odds ratio	95% CI	Wald	*p*-value
Health examination	1	—	—	—	1	—	—	—	1	—	—	—
Urogenital inflammation	1.02	0.77–1.35	0.01	0.913	1.55	1.26–1.90	17.36	< 0.001	2.27	1.26–4.07	7.53	0.006
Female infertility	0.49	0.36–0.66	22.33	< 0.001	0.09	0.07–0.13	204.24	< 0.001	0.19	0.08–0.43	15.83	< 0.001
Male infertility	0.17	0.11–0.27	52.83	< 0.001	0.14	0.10–0.20	108.51	< 0.001	0.04	0.01–0.30	9.7	0.002
History of adverse pregnancy and childbirth	0.44	0.27–0.73	9.96	0.002	0.11	0.06–0.21	44.89	< 0.001	0.06	0.00-0.94	4	0.045
Family planning guidance	1.19	0.80–1.77	0.7	0.402	0.95	0.69–1.31	0.09	0.713	0.57	0.19–1.77	0.94	0.332
other diagnoses	0.71	0.49–1.04	3.04	0.081	0.55	0.41–0.74	15.39	< 0.001	0.46	0.17–1.20	2.52	0.113

## Discussion

In this retrospective study, we investigated the prevalence of Chlamydia trachomatis (CT), Neisseria gonorrhoeae (NG), and Ureaplasma urealyticum (UU) among reproductive-age outpatients attending Shantou Central Hospital between 2023 and 2024—a population and setting that have received limited attention in the existing literature. Among the 13,622 patients analyzed, 11.14% tested positive for at least one pathogen. UU was the predominant pathogen in both sexes, with positivity rates of 48.97% in females and 27.37% in males. By contrast, CT and NG positivity rates were higher in males (5.49% and 15.01%, respectively) than in females (3.68% and 0.58%, respectively), and all sex-based differences were statistically significant. The majority of infections were attributable to a single pathogen; mixed infections were relatively uncommon. Collectively, these findings characterize the current epidemiological landscape of these three STI pathogens in a large reproductive-age outpatient cohort and provide a basis for informing targeted screening and prevention strategies in this population.

The infection rate of UU in our study (42.42%) was comparable to the data from Putian (48.81%) [15], Taizhou (62.04%) [16], and Changzhou (60.11%) [17], where high prevalence rates of UU have also been reported [18].This high prevalence is likely attributed to its natural colonization in healthy carriers, many of whom remain asymptomatic [19]. As a result, the pathogen is often undetected, allowing it to persist and spread within the population. Our study found that the infection rate of UU was significantly higher in females than in males, consistent with several previous studies [16, 20, 21]. Wang et al. reported the highest positivity rate of UU in the 21–50 age group, while Zheng et al. found that UU was most frequently detected in the 21–40 age group [18, 22]. These findings are likely linked to the sexually active status of these age groups. Therefore, when formulating public health policies for the prevention and control of UU, special attention should be given to the childbearing-age population.

The prevalence of CT in our study was slightly higher than the global average. A recent meta-analysis reports the global infection rate of CT in the general population as 2.9%, with females exhibiting a significantly higher infection rate (3.1%) than males (2.6%). In our study, the CT positivity rate in males was 5.49%, and in females, it was 3.68%, both exceeding the global average [23]. Notably, our study identified that the highest CT infection rate occurs in patients aged 25 and younger, regardless of sex, which is consistent with findings from studies conducted in Haikou [24], Putian [15], and Chongqing [25].

NG, the causative agent of gonorrhea, has a reported global prevalence of approximately 0.8% in the general population according to recent systematic reviews [26]. In our study, the prevalence among the childbearing-age population was 1.72%. Compared to other regions in China, this prevalence is higher than in Putian (0.23%) [15] and Chengdu (0.09%) [14], but lower than in Jingzhou (4.57%) [27]. Our study also found that the NG infection rate in males was 15.01%, significantly higher than in Chengdu (0.11%) [14] and Chongqing (0.12%) [25], but lower than in Kunming (23.11%) [28]. The variation in NG infection rates across regions may be influenced by factors such as economic conditions, cultural diversity, and lifestyle differences [29]. Additionally, the NG infection rate was notably higher in males than in females, likely due to the higher frequency of symptomatic infections in males, leading to more frequent diagnoses and treatments [30]. This disparity may also be influenced by behavioral and social factors, such as higher sexual risk behaviors and a greater number of sexual partners [31].

Our study demonstrated that mixed infections were uncommon, with CT + UU representing the most frequent co-infection pattern, accounting for 50% of all mixed infections. These findings are consistent with previous reports from Taizhou [16], Shanghai [32] and Chengdu [14]. Several biological mechanisms may underlie this frequent co-occurrence. CT is an obligate intracellular pathogen that infects epithelial cells, inducing cell lysis, pro-inflammatory cytokine production (e.g., IL-1β and IL-8), and epithelial barrier disruption [33, 34]. This compromised epithelial integrity may facilitate Ureaplasma colonization or ascension to the upper genital tract. Concurrently, U. urealyticum hydrolyzes urea to produce ammonia, thereby elevating local pH and potentially reducing colonization resistance, which may promote the establishment and persistence of co-infecting organisms [35]. Moreover, CT-induced inflammation shifts the local vaginal pH from its physiologically acidic range (~ 4.5) toward neutrality [36], potentially creating conditions favorable for Ureaplasma growth, which is known to thrive at pH 6.0–7.0 [37]. Nevertheless, whether these CT-mediated microenvironmental alterations act synergistically to enhance susceptibility to Ureaplasma co-infection in vivo remains to be established. Further mechanistic studies are warranted to elucidate the pathophysiological basis and clinical significance of this co-infection pattern.

Previous studies have demonstrated a strong association between CT and NG infections and urogenital tract inflammation [38]. In male urethral discharge samples, NG and CT are the most common pathogens responsible for STIs [39]. In females, these pathogens are also the principal etiologic agents of urogenital inflammation, including cervicitis and pelvic inflammatory disease, which may involve the uterus, fallopian tubes, ovaries, and/or pelvic peritoneum [40]. In contrast, our study identified a higher prevalence of UU among patients with urogenital inflammation. We also observed that infertility-related indications were generally associated with lower odds of pathogen positivity compared with routine health examination. Several factors may explain these findings. First, infertility clinics often include a large proportion of individuals undergoing screening rather than symptom-driven evaluation, which may yield lower detection rates for symptomatic pathogens such as NG. Second, infertility populations may differ systematically from routine examinees in age structure, sexual behavior, antibiotic exposure, and healthcare-seeking patterns, all of which can confound crude comparisons. Third, non-uniform testing across pathogens may further affect comparability if clinicians selectively ordered CT/UU/NG assays based on sex or clinical suspicion. Nevertheless, CT remains clinically relevant in infertility care given its established links to tubal factor infertility and adverse reproductive outcomes; therefore, targeted screening strategies in reproductive-age patients should be considered. Beyond the infertility population, our findings carry important public health implications. UU was detected in 11.50% of individuals undergoing routine physical examinations, and a notable proportion of NG-positive cases were identified within this otherwise asymptomatic group. The presence of active infection in the absence of clinical symptoms highlights the risk of unrecognized onward transmission and underscores the limitations of symptom-based testing strategies. Collectively, these findings support the broader implementation of routine STI screening among reproductive-age individuals to facilitate early detection, prompt treatment, and mitigation of silent transmission.

This study has several limitations. First, as a cross-sectional retrospective observational study, it does not allow for causal inference. Second, the marked female predominance (74% of study population) limits the generalizability of overall prevalence estimates and may underestimate the true burden of STIs in males, who often present only when symptomatic or may avoid testing due to stigma. This sex imbalance is a common limitation in reproductive health studies conducted in outpatient settings. Gender-specific prevalence estimates provide more meaningful data than overall prevalence in such circumstances. Future studies employing community-based or population-representative sampling would provide more accurate overall prevalence estimates. Additionally, the study population was heterogeneous, including both symptomatic and asymptomatic outpatients, as well as individuals with various sexually transmitted infections, which may limit the accuracy of prevalence estimates for the general reproductive-age population. Therefore, future targeted studies focusing on this broader demographic are warranted to obtain more precise and representative data. Furthermore, due to the constraints of existing medical records, this study could not assess other key risk factors, such as frequency of sexual activity, number of sexual partners, and socioeconomic status—factors that are critical for understanding and managing the transmission dynamics of sexually transmitted infections. Addressing these limitations in future research will be crucial to advancing our understanding of the epidemiology of STDs among individuals of reproductive age.

## Data Availability

The datasets generated and/or analysed during the current study are not publicly available due to the lack of an online platform but are available from the corresponding author on reasonable request.
